# Theoretical Insights into the Structures and Electronic Properties of Pure Germanium Anionic Ge*_n_*^−^ Clusters and Lanthanum-Doped Neutral and Anionic Germanium LaGe*_n_*^0/−^ Clusters (*n* = 10–20)

**DOI:** 10.3390/molecules31152679

**Published:** 2026-07-31

**Authors:** Xueyan Dong, Zhefeng Zhang, Chenliang Hao, Jucai Yang

**Affiliations:** 1 School of Chemical Engineering, Inner Mongolia University of Technology, Hohhot 010051, China; 2 School of Resources and Environmental Engineering, Inner Mongolia University of Technology, Hohhot 010051, China

**Keywords:** global minimum structures of Ge*_n_*^−^ and LaGe*_n_*^0/−^ clusters, configuration evolution, photoelectron spectra, UV–vis spectra, superatom

## Abstract

Doping provides an effective means to tailor the chemical properties of clusters and construct novel functional materials. However, the specific effects of rare-earth doping on the structural evolution and electronic properties of semiconductor clusters remain unclear. To address this, we systematically investigated the structures, growth patterns, electronic properties, and spectroscopic characteristics of Ge*_n_*^−^ and LaGe*_n_*^0/−^ clusters (*n* = 10–20) using the ABCluster global search method combined with the mPW2PLYP double-hybrid density functional. Notably, the global minimum (GM) structures of Ge*_n_*^−^ (*n* = 12–20), confirmed based on calculated energies and measured photoelectron spectroscopy data, differ from previously reported structures. Starting from *n* = 12, the GM structure of the Ge*_n_*^−^ cluster can be considered as formed by attaching an additional Ge_(*n*–9)_ or Ge_(*n*–10)_ subcluster to a *capped tetragonal antiprism* Ge_9_ (or *dicapped tetragonal antiprism* Ge_10_) subunit. The evolution pattern of LaGe*_n_* (*n* = 10–19) clusters can be viewed as substitutional structures, in which a La atom substitutes one Ge atom in the Ge_(*n*+1)_^−^ cluster. At *n* = 20, a cage-like structure is formed. For LaGe*_n_*^−^ (*n* = 10–19), when *n* = 10–12 and 18, the structures are linked configurations, where the La atom connects two Ge subclusters. For the remaining clusters, although their global minimum structures tend toward linked configurations, they are fundamentally substitutional in nature. The GM structure of LaGe_20_^−^ is an encapsulated configuration, with the La atom encapsulated at the center of the Ge cage. The average binding energies, relative stabilities, and HOMO–LUMO energy gaps of the clusters were evaluated. The photoelectron spectra of LaGe*_n_*^−^ (*n* = 10–20) and the UV–vis absorption spectrum of the LaGe_20_^−^ cluster were simulated. The results demonstrate that the LaGe_20_^−^ superatom cluster with high Ih symmetry exhibits favorable optical properties, along with excellent chemical and thermodynamic stability, suggesting its potential as a promising building block for further exploration in optoelectronic-related applications.

## 1. Introduction

Germanium clusters have garnered significant attention for their crucial roles in surface growth processes and electron transport [1,2,3,4,5,6,7,8]. However, pure germanium clusters exhibit high chemical reactivity, making them less suitable as building blocks for self-assembling materials [9,10,11,12,13,14,15,16]. The doping of nanoclusters is one of the most promising strategies in materials science. By selecting appropriate dopants, doping germanium clusters can modulate their structures and physicochemical properties, thereby enabling the design of novel functional materials. Known as the “vitamin of industry,” rare-earth elements possess unfilled 4*f* and/or 5*d* electrons, which not only neutralize the dangling bonds that cause instability in germanium clusters but also endow the resulting materials with exceptional magnetic, optical, electronic, and catalytic properties, making them widely applicable in electronics, optics, chemical engineering, energy, and other high-tech fields. As fundamental building blocks of nanomaterials, clusters provide a unique platform for investigating the dependence of physicochemical properties on structural evolution across different scales [17,18].

When the number of atoms in a system is relatively small (typically below 100), the influence of impurity atoms becomes particularly pronounced. Within this size regime, the properties of clusters exhibit strong dependence on their size, composition, and structure. Such characteristics enable researchers to design specialized functional materials through precise control of these parameters. Moreover, single rare-earth atom doping provides unusual advantages in controllability and directional design for optimizing the structural and electronic properties of clusters. Consequently, both theoretical and experimental investigations have been conducted on the structures and properties of rare-earth-doped germanium clusters. For instance, since Nakajima and coworkers utilized photoelectron spectroscopy and probe techniques to investigate the structural and electronic properties of rare-earth-doped germanium clusters (REGe*_n_*^−^ (RE = Sc, Y, Tb, Lu; *n* = 8–20), a number of scholars have employed theoretical methods to calculate their structures and properties, aiming to identify reliable computational approaches for predicting these features [19,20,21,22,23,24]. Both experimental and theoretical studies have demonstrated that the expanded cavity of the Ge16 cluster facilitates the formation of metalloid-encapsulated geometries, particularly in the Y@Ge_16_^−^ and Tb@Ge_16_^−^ clusters [19,22,23]. The most stable structures of ScGe*_n_*^−^ (*n* = 6–17), YGe*_n_*^−^ (*n* = 6–20), TbGe*_n_*^−^ (*n* = 6–17), and LuGe*_n_*^−^ (*_n_* = 5–17) were determined by comparing theoretical predictions with limited experimental data [21,22,23,24]. Furthermore, the structures, stability, photoelectron spectra, and other spectroscopic properties of germanium negative-ion clusters (Ge*_n_*^−^, *n* ≤ 20) doped with rare-earth atoms (Ce, Sm, Eu, Gd) were calculated using a double-hybrid density functional method. The results indicated that the minimum cluster sizes required for the formation of cage structures in Ce- and Sm-doped germanium negative ions are *n* = 16 and 20, respectively [16,18]. For Eu, even at *n* = 20, the ground-state structure does not adopt a cage configuration [17]. Research on rare-earth-doped neutral germanium clusters is relatively scarce compared to their doped anionic cluster counterparts [16,18].

In this study, pure germanium anionic clusters and lanthanum-doped neutral and anionic germanium clusters were selected as the research subjects, motivated by the unique electronic structure of lanthanum, which endows it with application potential in various fields such as hydrogen storage materials, cathode materials, alloy steels, lanthanide glasses, biological tracers, and cancer pain treatment. The incorporation of lanthanum atoms into the neutral and anionic germanium cluster system may impart novel properties, thereby enabling the construction of promising nanoscale multifunctional structural units. This study aims to determine the global minimum structures of pure germanium anionic Ge*_n_*^−^ and LaGe*_n_*^0/−^ (*n* = 10–20) clusters, investigate their structural evolution pattern, and simulate their spectroscopic properties. Additionally, the study will examine their electronic properties, charge transfer, HOMO-LUMO energy gaps, and relative stability, laying a theoretical foundation for the future synthesis and investigation of lanthanum-doped (or other rare-earth/transition metal-doped) semiconductor nanoclusters.

## 2. Results and Discussion

### 2.1. Global Minimum of Ge_n_^−^ and LaGe_n_^0/−^ (n = 10–20) Clusters

**Figure 1:**
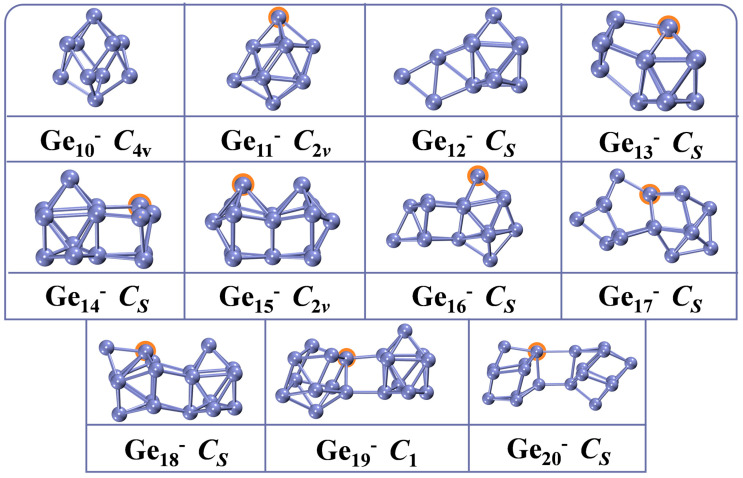
The GM structures of Ge*_n_^−^* (*n* = 10–20) clusters obtained at the mPW2PLYP level, where the orange circles represent the positions of substituted Ge atoms when La is doped.

**Figure 2:**
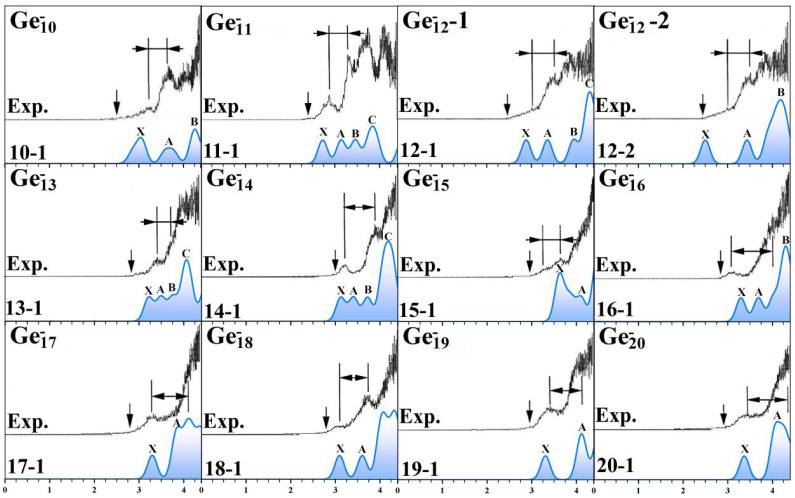
Theoretical and experimental photoelectron spectra of anion Ge*_n_*^−^ (*n* = 10–20) clusters. Experimental photoelectron spectra were taken from Ref. [6]. In the spectra, the first peak is denoted as X, and the remaining peaks are labeled A, B, C, and so forth in order of increasing energy.

Before discussing the doping of lanthanum atoms into germanium anionic clusters, let us first determine the global minimum (GM) structures of pure germanium anionic Ge*_n_*^−^ (*n* = 10–20) clusters. Regarding germanium anionic clusters, Tai et al. [1] first investigated the global minimum (GM) structures of Ge*_n_*^−^ (*n* = 2–12) using a stochastic search method. Later, in 2018, An Wei [2] computed the structures of Ge*_n_*^−^ (*n* = 3–20) using previously reported configurations from the literature as candidates for the ground-state structures. Very recently, Li et al. [15] reported the ground-state structures of Ge*_n_*^−^ (*n* = 4–15). Our results are shown in Figure 1. The electronic states of these structures are all doublets. These structures were identified through three approaches. First, among the isomers determined via the global search, the identified GM structures exhibited the lowest energies. Second, their calculated first VDEs are in good agreement with the experimental VDE values. Third, their simulated photoelectron spectra show good agreement with the experimental spectra in terms of both peak positions and numbers. For *n* = 12, the *C_s_*-symmetric structure reported here is energetically degenerate with the previously reported *C*_2*v*_-symmetric structure [1] at the mPW2PLYP level. Nevertheless, the simulated VDEs indicate that the *C_s_*-symmetric isomer yields a VDE value (2.88 eV) in better agreement with the experimental value (3.05 eV) [6] compared to the *C*_2*v*_ isomer (2.50 eV). Moreover, the simulated photoelectron spectrum of the *C_s_*-symmetric structure matches well with the experimental spectrum in both peak positions and numbers, whereas that of the *C*_2*v*_-symmetric structure does not (see Figure 2). Consequently, the GM structure is assigned to the *C_s_*-symmetric one. The calculated and experimental VDE values for the Ge*_n_*^−^ (*n* = 10–20) clusters are listed in Table 1. Table 1 shows that the average deviation between the two is only 0.09 eV. In simulating the peak positions and numbers, although the experimental spectra [5,6] exhibit relatively smooth features with no pronounced peaks, the simulated spectra are in good overall agreement with the experimental ones. In summary, the GM structures of Ge*_n_*^−^ (*n* = 10–20) reported in this work are reliable. It should be noted that, except for *n* = 10 and 11, the GM structures reported in this work differ from those reported in the aforementioned studies. From Figure 1, we can draw the following conclusion: starting from *n* = 12, the GM structure of the anionic Ge*_n_*^−^ cluster can be considered as formed by attaching an additional Ge_(*n*–9)_ or Ge_(*n*–10)_ subcluster to a *capped tetragonal antiprism* Ge_9_ (or *dicapped tetragonal antiprism* Ge_10_) subunit.

**Figure 3:**
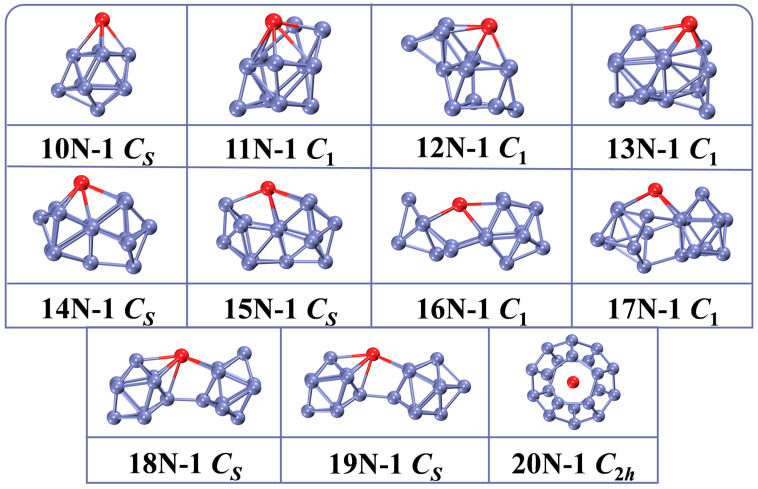
The GM structures of LaGe*_n_* (*n* = 10–20) clusters obtained at the mPW2PLYP level. Red and blue denote La and Ge atoms, respectively.

**Figure 4:**
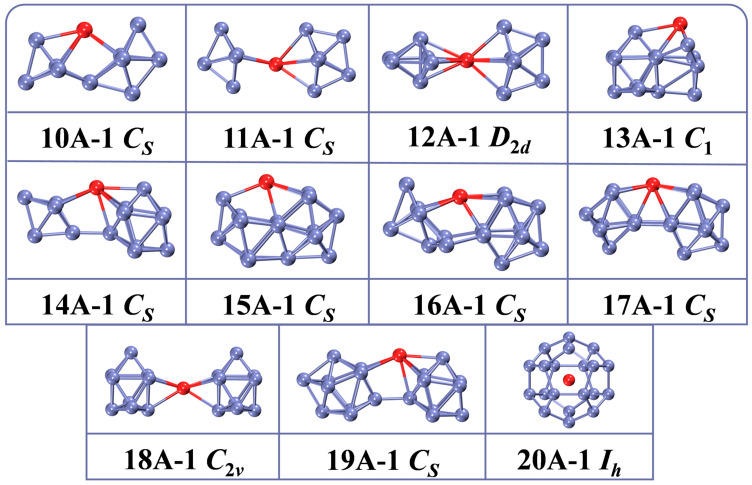
The GM structures of LaGe*_n_*^−^ (*n* = 10–20) clusters obtained at the mPW2PLYP level. Red and blue denote La and Ge atoms, respectively.

Figure 3 and Figure 4 show the GM structures of LaGe*_n_*^0/−^ (*n* = 10–20) clusters, respectively. The structures, energies, and symmetries of the corresponding low-lying isomers are shown in Figures S1 and S2 in the Supplementary Materials. Table 2 summarizes the electronic states, binding energies, and NPA charges on the La atom of the GM structures. The examined spin states for the neutral and anionic clusters are singlets and doublets, respectively. The GM structures for neutral LaGe*_n_* (*n* = 10–19) clusters can be regarded as being derived from the GM geometry of anionic Ge*_n_*_+1_^−^ by replacing a Ge atom with a La atom, denoted as substitutional structures. Their GM structures can be described as follows: an anionic Ge_11_^−^ *tricapped tetragonal antiprism*; Ge_12_^−^ formed by *triangle* Ge_3_ addition to Ge_9_; Ge_13_^−^ formed by *roughly rhombus* Ge_4_ addition to Ge_9_; Ge_14_^−^ formed by *distorted tetragonal monopyramid* Ge_5_ addition to Ge_9_; Ge_15_^−^ formed by *pentagonal monopyramid* Ge_6_ addition to Ge_9_; Ge_16_^−^ formed by *bicapped tetragonal monopyramid* Ge_6_ addition to Ge_10_; Ge_17_^−^ formed by *distorted bicapped pentagonal monopyramid* Ge_7_ addition to Ge_10_; Ge_18_^−^ formed by Ge_9_ addition to Ge_9_; Ge_19_^−^ formed by Ge_9_ addition to Ge_10_; and Ge_20_^−^ formed by Ge_10_ addition to Ge_10_ with one Ge atom replaced by a La atom (see Figure 1). Since the La atom has a larger diameter than the Ge atom, the structure of the LaGe*_n_* cluster undergoes significant deformation compared with that of the Ge*_n_*_+1_^−^ cluster after substitution. For the LaGe_20_ cluster, its GM structure is an encapsulated configuration with a La atom in the germanium cage.

For *n* = 10–12 and 18, their GM structures are linked configurations. The La atom in LaGe_10_^−^ links two *perpendicular trigonal-bipyramidal* Ge_5_ subclusters. In LaGe_11_^−^, the La atom links a *trigonal-bipyramidal* Ge_5_ and a *distorted tetragonal-bipyramidal* Ge_6_ subcluster. In LaGe_12_^−^, the La atom links two perpendicular *distorted tetragonal-bipyramidal* Ge_6_ subclusters. In LaGe_18_^−^, the La atom links two parallel *capped tetragonal antiprism* Ge_9_ subclusters. For *n* = 13–17 and 19, despite the tendency of their GM structures toward linked configurations, they are fundamentally substitutional in nature. For the LaGe_20_^−^ cluster, its GM structure is an encapsulated configuration with the La atom in the Ge cage.

**Figure 5:**
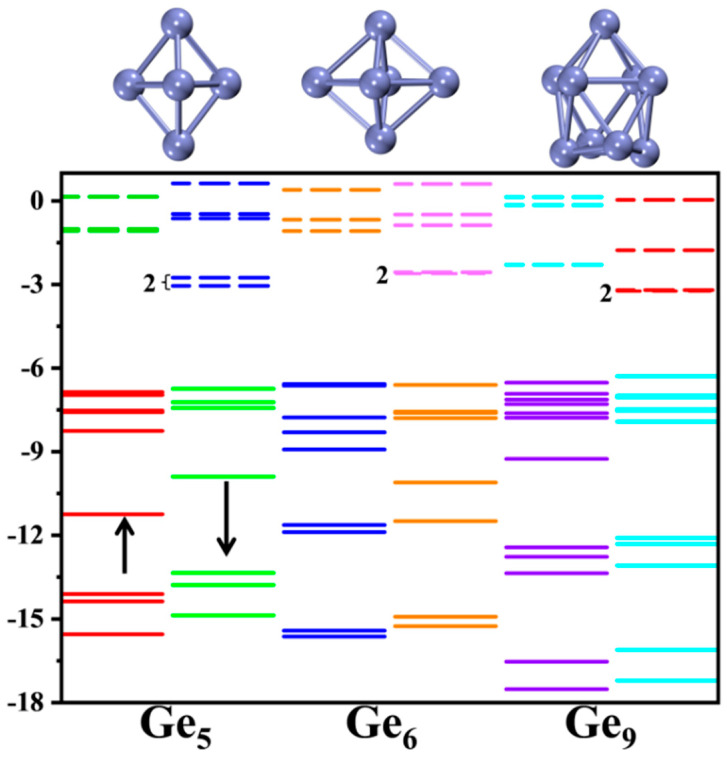
(Color online) mPW2PLYP eigenstates of Ge5, Ge6, and Ge9 clusters that are found to exist in linked configurations are shown. Broken lines show unoccupied states. The numbers 2, 2, and 2 indicate the number of unoccupied states in the down-spin spectra. The geometries are shown above the energy spectra. The arrows show up-spin and down-spin spectra.

The effect of extra electrons on the configurations is evident, and compared with the neutral configurations, the anionic configurations undergo significant changes. For instance, for *n* = 10–12 and 18, the formation of stable linked structures can be explained by the spin-polarized electronic structures of subclusters such as Ge_5_, Ge_6_, and Ge_9_ (similar to those of Si_5_, Si_6_, and Si_9_ [25]). As shown in Figure 5, the up-spin spectrum of the *trigonal bipyramid* Ge_5_ has a large energy gap between the highest and lowest unoccupied states, within which there are two unoccupied states in the down-spin spectrum. This indicates that this cluster requires two electrons to achieve stability. Since Lu^−^ has four valence electrons, it can connect two clusters like Ge_5_ in such a way that each cluster has two holes in its highest occupied molecular orbital (HOMO). In the LaGe_10_^−^-linked configuration, Lu^−^ provides (or shares) four electrons (two electrons to each *trigonal bipyramid* Ge_5_), thereby stabilizing **10A-1**. For the *tetragonal bipyramid* Ge_6_ and the *capped tetragonal antiprism* Ge_9_, their spin-polarized electronic structures exhibit features similar to those of Ge_5_. Therefore, in the **11A-1**-linked geometry, two electrons from Lu^−^ are shared with the germanium *trigonal bipyramid* and *tetragonal bipyramid*, respectively; in the **18A-1**-linked geometry, each *capped tetragonal antiprism* shares two electrons with Lu^−^, thereby stabilizing the cluster.

### 2.2. Simulated PES of LaGe_n_^−^ (n = 10–20) Clusters

**Figure 6:**
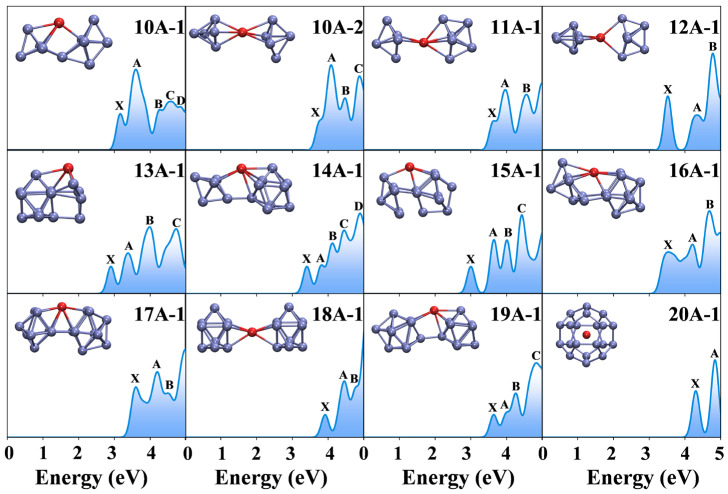
Simulated photoelectron spectra of the ground-state structures of LaGe*_n_*^−^ (*n* = 10–20) clusters. In the spectra, the first peak is denoted as X, and the remaining peaks are labeled A, B, C, and so forth in order of increasing energy.

Photoelectron spectroscopy (PES) is not merely a characterization tool; rather, it serves as a cornerstone for understanding the electronic structure and chemical stability of matter, because it can sensitively detect structural changes through variations in peak shape, peak position, and peak number. In this study, the PES spectra of the ground-state structures of LaGe*_n_*^−^ (*n* = 10–20) were simulated at the mPW2PLYP level based on Koopmans’ theorem, with the full width at half maximum (FWHM) set to 0.25 eV. The energy range was selected as 0–5 eV. The final results are shown in Figure 6. In the spectra, the first peak is denoted as X, and the remaining peaks are labeled A, B, C, and so forth in order of increasing energy. Because nonadiabatic interactions and anharmonic resonances were not included in the calculations, the peak intensities along the vertical axis are not quantitatively comparable.

For **10A-1**, the simulated PES shows four prominent peaks (X, A, B, C) centered at 3.82, 4.08, 4.47 and 4.87 eV in the low binding energy range of ≤5.0 eV, and peak X appears as a shoulder of peak A. For **11A-1**, the simulated PES has three obvious peaks (X, A, B) centered at 3.65, 3.96, and 4.56 eV, and peak X appears as a shoulder of peak A, similar to **10A-1**. For **12A-1**, three peaks (X, A, B) are observed at 3.52, 4.34, and 4.78 eV, respectively. For *n* = 13–15, multiple distinct and successive peaks are observed. In **13A-1**, four peaks are assigned to X (2.90 eV), A (3.38 eV), B (3.98 eV), and C (4.73 eV), with increasing intensities. In **14A-1**, five peaks appear at X (3.41 eV), A (3.82 eV), B (4.12 eV), C (4.44 eV), and D (4.90 eV); in addition to the isolated X peak, the others also show increasing intensities. In **15A-1**, peak X is isolated at 3.00 eV, while peaks A (3.65 eV), B (4.01 eV), and C (4.43 eV) are successive with similar adjacent energy separations. Overall, these three cases share comparable peak profiles and positions, warranting careful experimental distinction. For **16A-1**, within the selected energy range, the number of peaks is markedly reduced, with only a broad, smooth X peak at 3.54 eV, a weak A peak at 4.21 eV, and an isolated B peak at 4.68 eV. For **17A-1**, peak X lies at 3.60 eV, while peak A (4.20 eV) is smoothly connected to it; peak B (4.49 eV) appears as a small shoulder to the right of A. For **18A-1**, the three PES peaks are closely spaced and assigned to X (3.92 eV), A (4.45 eV), and B (4.80 eV). For **19A-1**, four peaks are observed: X (3.64 eV), B (4.26 eV), C (4.83 eV), and peak A (3.99 eV) as a shoulder of B. For **20A-1**, which has a highly symmetric cage-like structure, the PES shows two well-resolved peaks X and A at 4.30 and 4.84 eV. Given the lack of experimental data, the simulated spectra and peak positions presented here offer a useful reference for future experiments.

In addition to PES, the adiabatic electron affinity (AEA, defined as the difference in total energies in the manner AEA = E(optimized neutral GM) − E(optimized anion GM)) is another key parameter for assessing the stability and reactivity of substances. Acting as a link between the microscopic electronic structure and the macroscopic chemical stability and electrochemical behavior, the AEA provides crucial insights. The VDE–AEA gap, known as the reorganization energy, offers a direct quantitative measure of the geometric distortion occurring during ionization; a larger gap indicates a greater nuclear displacement upon neutralization of the anion. Table 3 lists the VDE and AEA values for LaGe*_n_*^−^ (*n* = 10–20). It is evident that for *n* = 10–12, 17, and 18, the VDE–AEA differences exceed 0.5 eV. For *n* = 10–12 and 18, as previously noted, the large distortion originates from the structural transformation from a linked anion to a substituted neutral. For *n* = 17, the Ge_8_ subunit shows substantial nuclear displacement, changing from a *tetragonal antiprism* in the anion to a *distorted cube* in the neutral state. For *n* = 14, the VDE–AEA gap is about 0.43 eV, suggesting that the Ge_5_ subcluster distorts from a *trigonal bipyramid* in the anion to a *distorted tetragonal monopyramid* in the neutral state. For the remaining clusters (*n* = 13, 15, 16, 19, 20), the smaller VDE–AEA gaps indicate only minor nuclear displacements upon neutralization. In the absence of experimental values for comparison, and considering that anion PES is a well-established technique for determining AEA, it is nonetheless challenging to extract accurate experimental AEA values when the spectrum displays a long, smooth tail without pronounced features. Thus, the AEA values reported in this work provide an important theoretical benchmark for experimental measurements.

### 2.3. The Relative and Chemical Stability of Ge_n_^−^ and LaGe_n_^0/−^ (n = 10–20) Clusters

The average binding energy (*ABE*) and HOMO-LUMO energy gap (*E*_gap_) are two important parameters for evaluating cluster stability, corresponding to thermodynamic and relative stability and chemical stability, respectively. In this study, these two parameters were calculated for Ge*_n_*^−^ and LaGe*_n_*^0/−^ (*n* = 10–20) clusters, and the corresponding formulas are given as follows:

(1) $$ABE({\mathrm{Ge}}_{n}^{-})=\frac{\left(n-1)E(\mathrm{Ge})+E({\mathrm{Ge}}^{-})-E({\mathrm{Ge}}_{n}^{-}\right)}{n}$$

(2) $$ABE({\mathrm{LaGe}}_{n}^{0})=\frac{nE(\mathrm{Ge})+E(\mathrm{La})-E({\mathrm{LaGe}}_{n}^{0})}{n+1}$$

(3) $$ABE({\mathrm{LaGe}}_{n}^{-})=\frac{\left(n-1)E(\mathrm{Ge})+E({\mathrm{Ge}}^{-})+E(\mathrm{La})-E({\mathrm{LaGe}}_{n}^{-}\right)}{n+1}$$

(4) $$E_{\mathrm{gap}}=E(\mathrm{LUMO})-E(\mathrm{HOMO})$$

**Figure 7:**
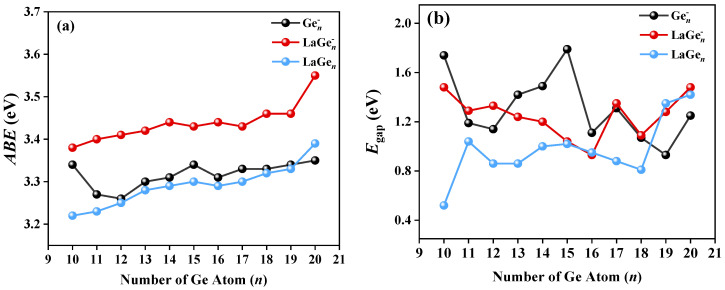
Size dependences of (**a**) average bonding energy (*ABE*) calculated at the mPW2PLYP level and (**b**) HOMO–LUMO energy gap (*Egap*) calculated at the PBE level for the GM structure of LaGe*_n_*^−^ (*n* = 10–20) species.

Figure 7a presents the *ABE* values for Ge*_n_*^−^ and LaGe*_n_*^0*/*−^ (*n* = 10–20) clusters and simultaneously illustrates the evolution of *ABE* as a function of successive Ge atom addition. A higher *ABE* value corresponds to enhanced cluster stability. As revealed in the figure, the following observations can be made: (1) For *n* = 10–19, the incorporation of an additional electron into a germanium cluster confers greater stability than the interstitial doping of a lanthanum atom. For *n* = 20, however, the LaGe*_n_* cluster is slightly more stable than the Ge*_n_*^−^ cluster due to the formation of a cage-like structure. Moreover, as one would expect, the pristine germanium anionic clusters are stabilized upon single lanthanum atom doping. (2) Starting from *n* = 12, their relative stability exhibits an approximately parity-dependent behavior. For the LaGe*_n_*^−^ clusters, the even-*n* species at *n* = 14, 16, 18, and 20 are relatively more stable than the odd-*n* ones at *n* = 13, 15, 17, and 19. In contrast, for both pure germanium anionic clusters and neutral LaGe*_n_* clusters, the opposite holds true: the odd-*n* species (*n* = 13, 15, 17, 19) are relatively more stable than their neighboring clusters. (3) LaGe_20_^−^, characterized by a highly symmetric cage-like configuration, exhibits the highest thermodynamic stability among the clusters in this series.

The HOMO–LUMO energy gap (*E*_gap_) is an important physical parameter. To some extent, it can reflect the chemical reactivity of clusters, particularly for transition-metal-doped semiconductor clusters that may exhibit good photochemical reactivity. The *E*_gap_ calculated using pure density functional theory is closer to the true optical energy gap than that evaluated via hybrid density functionals. This is because, within the Kohn–Sham molecular orbital approximation, the predicted HOMO and LUMO levels generally shift upward by similar magnitudes, whereas the Hartree–Fock method shifts the LUMO to a much higher energy level than the HOMO [26]. Therefore, in this work, the *E*_gap_ values of the clusters were calculated using the PBE pure density functional [27] in conjunction with the def2-TZVPPD basis sets for La and Ge atoms, and the results are presented in Figure 7b. Meanwhile, as can be seen from Figure 7b, these clusters do not exhibit an odd–even oscillation behavior. The *E*_gap_ values are relatively large for Ge_10_^−^ and Ge_15_^−^, being 1.74 and 1.79 eV, respectively, while the smallest value is found for LaGe_10_, at 0.52 eV. The *E*_gap_ values of LaGe*_n_*^−^ (*n* = 10–20) generally fall within an intermediate range, from 0.93 to 1.48 eV, indicating that they possess good chemical activity and are beneficial for the preparation of novel functional materials, such as optical or optoelectronic photosensitive materials. For LaGe_20_^−^, its *E*_gap_ is close to that of the neutral counterpart, suggesting that the extra electron does not alter its chemical reactivity, but enhances its thermodynamic stability.

### 2.4. Natural Population Analysis of the LaGe_n_^0/−^ (n = 10–20) Clusters

To further understand the interaction between the La atom and the germanium clusters, natural population analysis (NPA) was performed on LaGe*_n_*^0*/*−^ (*n* = 10–20) clusters at the mPW2PLYP level. The charges of the La atom are given in Table 1, and its valence electron distributions are provided in Table S2 (see the Supplementary Materials). The valence electron configuration is [core]6*s*^0.21−0.31^4*f*^0.03−0.05^5*d*^1.45−1.74^6*p*^0.13−0.42^ ([core]6*s*^0.31^4*f*^0.03^5*d*^2.96^6*p*^1.07^) for the neutral clusters and [core]6*s*^0.22−0.30^4*f*^0.03−0.04^5*d*^1.42−1.77^6*p*^0.26−0.49^ ([core]6*s*^0.30^4*f*^0.03^5*d*^2.91^6*p*^1.06^) for anionic clusters with *n* = 10–19 (and *n* = 20). This indicates that the charge transfer of La occurs predominantly between the 6*s* and 5*d* and/or 6*p* orbitals, resulting in hybridization between the 6*s* and 5*d* (or 6*p*) orbitals. The charges of La in the system are 0.67–0.94 a.u. and 0.51–0.87 a.u. for the neutral molecules and anions of the non-cage structures, respectively, indicating that the La atom acts as an electron donor, and that the characteristics of bonding between La and the germanium clusters possess not only ionic bonds but also covalent bonds. The majority of the extra electron’s charge is localized on the germanium clusters. Compared to the corresponding neutral species, an average of 0.17 a.u. of charge flows back to the La atom from the germanium clusters. As a result, the ionic bond components are decreased, while the covalent bond components are increased, making the anionic clusters more stable than their corresponding neutral counterparts. For *n* = 20, the charges are −1.70 a.u. and −1.62 a.u., respectively, indicating that the La atom acts as an electron acceptor and forms an electron gas at the center of the cage. The reasons why the anionic clusters are more stable than their corresponding neutral counterparts will be explained below.

### 2.5. Chemical Bonding Analysis for Superatom LaGe_20_^−^ Cluster

**Figure 8:**
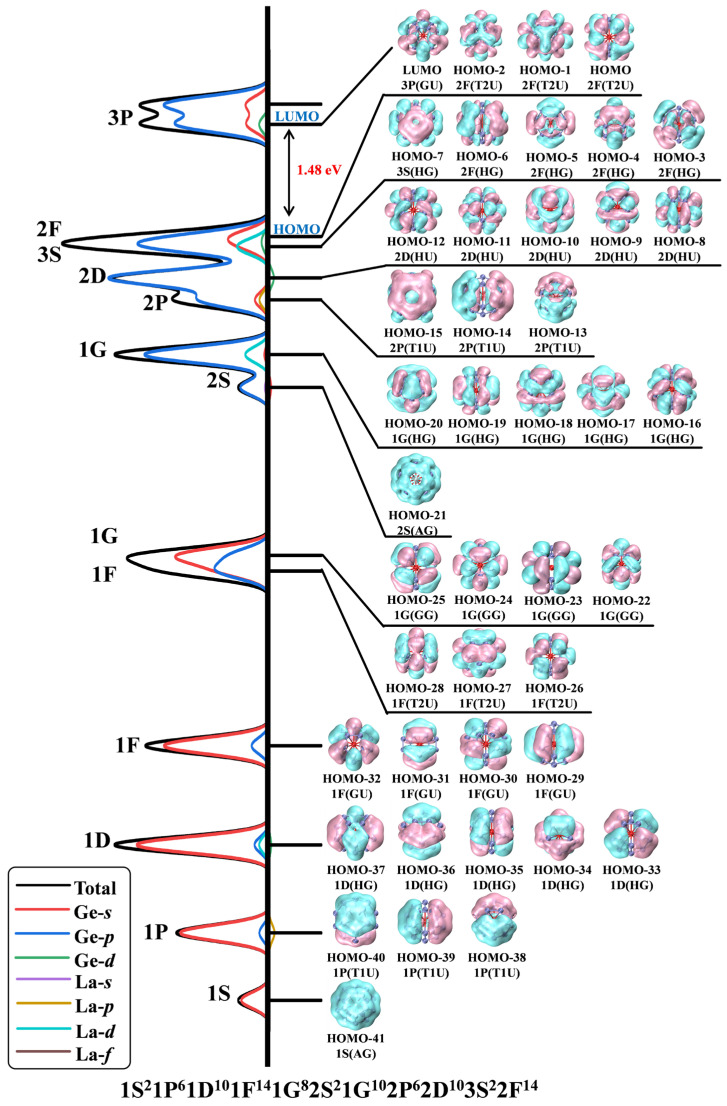
The molecular orbitals (MOs) and density of state (DOS) of the LaGe_20_^−^ cluster.

**Figure 9:**
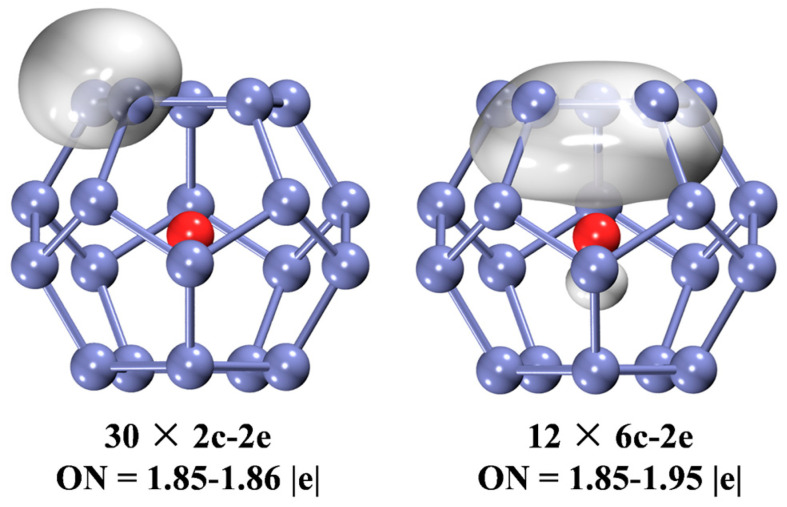
AdNDP analysis of the LaGe_20_^−^ cluster; ON stands for the occupation number.

To gain an in-depth understanding of the favorable thermodynamic stability and chemical reactivity of the anionic LaGe_20_^−^ cluster, we first analyze its molecular orbitals (MOs) and their energies based on the spherical jellium model. As shown in Figure 8 (MO and density of states (DOSs) diagrams of the LaGe_20_^−^ cluster), the electronic shells for the superatom LaGe_20_^−^ with the magic number of 84 valence electrons are best described as 1S^2^1P^6^1D^10^1F^14^1G^8^2S^2^1G^10^2P^6^2D^10^3S^2^2F^14^. In this configuration, the 1S jellium model shell features *s* bonds between the *s* orbitals of Ge atoms and the *s* orbital of the central La atom. The 2S shell features *sp* bonds between the *s* orbital of the central La atom and the *p* orbitals of Ge atoms. The 3S shell features *spd* bonds between the *d* orbital of the central La atom and the *sp* orbitals of Ge atoms. Both the 1P and 2P jellium model shells are threefold degenerate; the 1P shell primarily characterized by Ge 3*s* and La 5*p* orbitals, while the 2P shell is mainly characterized by La 5*p* and Ge 3*s*3*p* orbitals. Both the 1D and 2D jellium model shells are fivefold degenerate; the 1D shell is mainly formed by *sp* bonds from the *s* and *p* orbitals of Ge atoms, whereas the 2D shell is characterized by *pd* bonds formed by the *p* orbitals of Ge atoms and the *d* orbitals of La atoms. The 1F and the first four orbitals of the 1G shells (HOMO-22 to HOMO-25), similar to the 1D shell, are mainly formed by *sp* bonds from the *s* and *p* orbitals of Ge atoms, while the last five orbitals of the 1G shell and the 2F shell are characterized by *spd* bonds formed by the *sp* orbitals of Ge atoms and the *d* orbitals of La atoms. Overall, the frontier orbital region exhibits dense energy levels and significant La–Ge orbital hybridization. The degeneracy of the highly occupied orbitals may be attributed to the high symmetry *I_h_* of the LaGe_20_^−^ cluster. The 5*d* orbitals of Ge atoms contribute significantly to the occupied MOs and enhance the interactions between the outer fullerene Ge_20_ cage and the central La atom. Together, the DOS and MO analyses confirm that LaGe_20_^−^ can be regarded as a cluster with pronounced superatom characteristics. In addition, the AdNDP method was employed to perform a quantitative analysis of the nature of the bonding between the La atom and the Ge_20_ shell. As shown in Figure 9, the chemical bonding of the 84 valence electrons can be classified into two types: 2c-2e and 6c-2e bonds. The Ge_20_ cage is characterized by thirty localized 2c-2e and Ge-Ge bonds with 1.85–1.86 |e| in each bond. The twelve delocalized 6c-2e bonds (with 1.85–1.95 |e| in each bond) are responsible for the conjugation between the central La atom and the outer fullerene shell of Ge_20_, and stabilize the encapsulated LaGe_20_^−^ cluster.

### 2.6. UV–Vis Spectra of the Superatom LaGe_20_^−^ Clusters

**Figure 10:**
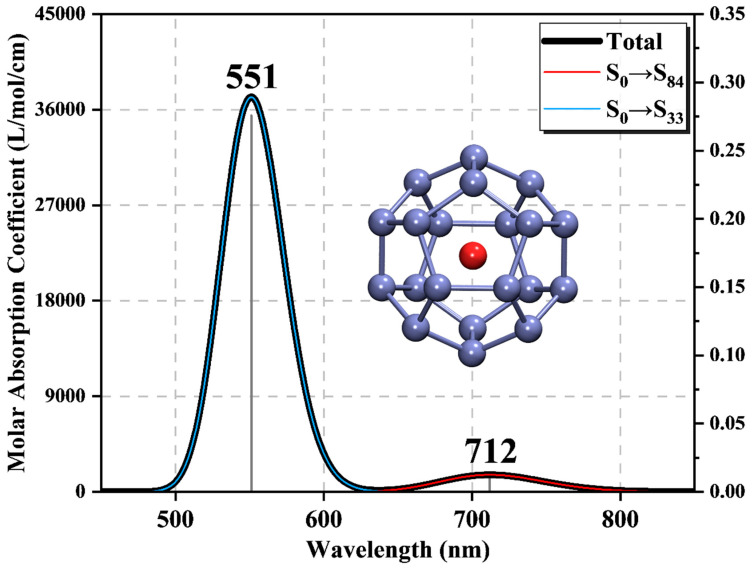
The UV–vis absorption spectrum of the LaGe_20_^−^ cluster.

Given the unique geometric structure and favorable stability of the LaGe_20_^−^ cluster, we further investigated its optical properties. The ultraviolet–visible absorption spectrum (UV–vis) of this cluster was simulated at the time-dependent density functional theory (TD-DFT) level [28]. A total of 120 excited states were calculated to ensure adequate coverage of the main absorption features within the target spectral range, and the full width at half maximum was set to 0.25 eV. The simulated UV–vis spectrum is shown in Figure 10. As shown in the figure, the LaGe_20_^−^ cluster exhibits two prominent absorption peaks. The first absorption peak, located at 551 nm, mainly arises from three nearly degenerate transitions, S_0_→S_82_, S_0_→S_83_ and S_0_→S_84_, with contributions of 33.3%, 33.4% and 33.3%, respectively. Here, “S” denotes a singlet state. The second absorption peak appears at 712 nm and is composed of three nearly degenerate transitions, S_0_→S_32_, S_0_→S_33_ and S_0_→S_34_, with corresponding contributions of 33.6%, 32.8% and 33.6%, respectively. Notably, the simulated absorption spectrum lies almost entirely within the visible-light region (380–780 nm) and can be excited by natural light, particularly with solar energy as the energy source for driving photosensitive or optoelectronic devices. In summary, the superatom LaGe_20_^−^ cluster with high *I_h_* symmetry not only possesses favorable optical properties but also exhibits excellent chemical and thermodynamic stability, making it the most suitable building block for further development as a potential photosensitive or optoelectronic device.

**Figure 11:**
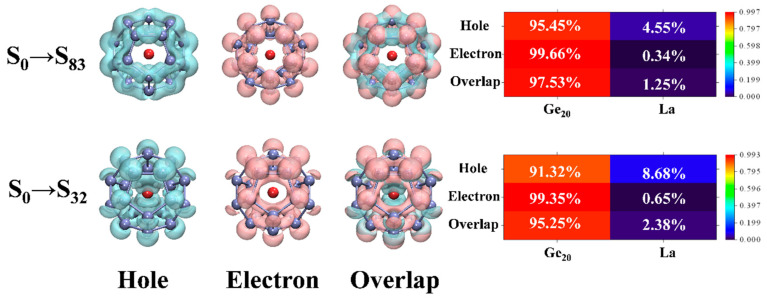
The hole–electron isosurfaces and heatmap of crucial excited states of the LaGe_20_^−^ cluster. The light blue and pink regions represent the spatial distributions of holes and electrons before and after excitation, respectively. In the heatmaps, the color changes from blue to red, indicating increasing contributions of the hole, electron, and hole–electron overlap in each fragment.

On the basis of the favorable optical response of the LaGe_20_^−^ cluster, its electronic excitation behavior was further investigated using the electron–hole analysis method developed by Liu et al. [29]. The S_0_→S_83_ transition from the first absorption peak and the S_0_→S_32_ transition from the second absorption peak were selected as representative excitation processes. The corresponding electron–hole distribution maps and electron–hole heatmaps were plotted, as shown in Figure 11. In the electron–hole distribution maps, the blue isosurfaces represent the “holes,” corresponding to the spatial distribution of electrons before excitation, whereas the pink isosurfaces represent the “electrons” corresponding to the spatial distribution after excitation. The electron–hole heatmaps quantitatively characterize the distribution and transfer of electrons and holes among different structural fragments during excitation. In this work, the Ge_20_ framework is defined as fragment 1, and the central La atom is defined as fragment 2. For the S_0_→S_83_ transition, the electrons before excitation are mainly distributed over the Ge–Ge bonding regions, whereas after excitation they tend to be localized around the Ge atoms. Meanwhile, almost no obvious electron or hole distribution is observed on or around the La atom before or after excitation. The heatmap analysis shows that the electron–hole overlap within fragment 1 reaches 97.53%, indicating that this transition is dominated by localized excitation (LE). The excitation process mainly occurs within the Ge_20_ framework and essentially corresponds to electron-density redistribution inside the Ge_20_ outer cage. In contrast, the S_0_→S_32_ transition exhibits more complex electron-transfer behavior. Before excitation, electron density is distributed not only over the Ge_20_ framework but also around the La atom. After excitation, the electron density around La decreases markedly, while electron density is transferred toward the outer Ge framework, suggesting electronic coupling between the La atom and the Ge cage. For this transition, the electron–hole overlap within fragment 1 is also substantial, reaching 95.25%, indicating that this excitation is likewise primarily characterized as LE. The high electron–hole overlap suggests that the LaGe_20_^−^ cluster may have potential applications as a visible-light-absorbing material, optical sensing material, or luminescent or photoresponsive functional unit.

## 3. Computational Strategy

The computational methodology has been described in detail in previous publications [16,17,18,19,20,21,22,23,24]; only a concise overview is provided here. The initial structures were determined using four approaches: (i) global geometry optimization via the ABCluster (Artificial Bee Colony algorithm for clusters) [30,31,32] technique in conjunction with the Gaussian 09 software package [33]; for each cluster, 150 configurations were randomly generated by ABCluster for clusters containing fewer than nine atoms, while 400 configurations were generated for those with nine or more atoms; (ii) the method of replacing a Ge atom in the ground-state structures of anionic Ge*_n_*_+1_^−^ cluster with a La atom; (iii) previously reported global minimum structures of rare-earth-doped semiconductor clusters [16,17,18,19,20,21,22,23,24]; and (iv) manually designed architectures. Although global-search algorithms are efficient, they are essentially mathematical in nature. While they work well for small clusters, they cannot reach “ergodic” sampling on the potential energy surface for large sized clusters—especially for heteroatomic systems—through computational simulation alone. Therefore, manual design of structures guided by “certain patterns” serves as an important complementary strategy. For the initial structure searches, the single-hybrid B3LYP functional [34,35] was employed together with LanL2DZ basis sets [36] for Ge and La atoms. Subsequently, low-energy isomers with energies within 0.8 eV of the global minimum were then selected and re-optimized using B3LYP with def2-TZVP basis sets. For the def2-TZVP basis set, La was treated with an effective core potential that includes scalar-relativistic effects and replaces 46 core electrons [36]. Frequency calculations were performed at the same level; the absence of imaginary frequencies confirmed that the resulting geometries correspond to local minima on the potential energy surface. After completing the single hybrid density functional theory (DFT) step, the most stable candidates were once again chosen and re-optimized with the double-hybrid functional mPW2PLYP [37] and def2-TZVP basis sets [36]. Frequency calculations were not carried out at the mPW2PLYP level due to the limited computational capacity of our computing stations and time considerations. It is worth noting that the computational cost of frequency calculations at the mPW2PLYP level is comparable to that of MP2 calculations. Finally, single-point energy calculations were carried out at the mPW2PLYP level using a large basis set def2-TZVPPD for La and Ge atoms [36] to refine the total energies. The convergence criteria for the optimizations are as follows: maximum force (threshold: 0.000450), root mean square force (threshold: 0.000300), maximum displacement (threshold: 0.001800), root mean square displacement (threshold: 0.001200) and convergence criterion (10^−6^ a.u.). During the optimization, we took into account the doublet and quartet states for the neutral clusters, and the singlet and triplet states for the anionic clusters. However, the quartet state lies higher in energy than the doublet state, and the triplet state lies higher than the singlet state. To gain deeper insight into the interaction between the La atom and Gen0/^−^ clusters, natural population analysis (NPA) was performed at the mPW2PLYP/def2-TZVPPD level. The photoelectron spectra of LaGen^−^ (*n* = 10–20) clusters were simulated on the basis of Koopmans’ theorem. UV–vis absorption spectra were obtained via time-dependent DFT (TD-DFT) with the B3LYP functional and def2-TZVPPD basis sets [36]. All visualizations were generated using the Multifwn 3.3.7(dev) [38,39] and VMD 1.9.4 software packages [40]. All calculations were performed with the Gaussian 09 program suite [33].

By employing DFT for short-range interactions and a combination of HF exchange and MP2 correlation for long-range interactions, the double-hybrid mPW2PLYP method adequately incorporates weak interaction effects, which underpins its reliability for calculating the structures and properties of rare-earth-metal-doped semiconductors. For structural predictions, the ground-state geometries of ScSi*_n_*^0/−^ (*n* = 4–9), CeGe*_n_*^−^ (*n* = 5–17) and ScGe*_n_*^−^ (*n* = 6–17) determined by the mPW2PLYP method are in good agreement with those from ROCCSD(T) or DLPNO-CCSD(T) benchmarks [18,21,41]. Regarding VDE predictions, the average deviation between the mPW2PLYP-calculated VDEs and the experimental measurements for the 35 clusters (ScGe*_n_*^−^ (*n* = 6–17), YGe*_n_*^−^ (*n* = 12–20), TbGe_16_^−^, and LuGe*_n_*^−^ (*n* = 5–17), excluding LuGe_8_ and LuGe_11_) is merely 0.08 eV [21,22,23,24]. Additionally, the predicted VDEs of Ge*_n_*^−^ (*n* = 10–20) agree with the experimental values with an average deviation of 0.09 eV.

## 4. Conclusions

Using the ABCluster structure searching method combined with the mPW2PLYP double-hybrid density functional, the structures, growth patterns, electronic properties, and spectroscopic characteristics of pure anionic germanium Ge*_n_*^−^ clusters and lanthanum-doped neutral and anionic germanium LaGe*_n_*^0/−^ clusters (*n* = 10–20) were systematically investigated. Based on the calculated energies and the measured photoelectron spectroscopy data, the ground-state structures of Ge*_n_*^−^ were confirmed, with those for *n* = 12–20 differing from previously reported structures. Starting from *n* = 12, the global minimum structure of the anionic Ge*_n_*^−^ cluster can be considered as formed by attaching an additional Ge_(*n*–9)_ or Ge_(*n*–10)_ subcluster to a *capped tetragonal antiprism* Ge_9_ (or *dicapped tetragonal antiprism* Ge_10_) subunit. The evolution pattern of neutral LaGe*_n_* (*n* = 10–19) clusters can be viewed as substitutional structures, in which a La atom substitutes for one Ge atom in the anionic Ge_(*n*+1)_^−^ cluster. At *n* = 20, a cage-like structure is formed. For anionic LaGe*_n_*^−^ (*n* = 10–19), when *n* = 10–12 and 18, the structures are linked configurations, where the La atom connects two Ge subclusters. For the remaining clusters, although their global minimum structures tend toward linked configurations, they are fundamentally substitutional in nature. The global minimum structure of LaGe_20_^−^ is an encapsulated configuration, with the La atom encapsulated at the center of the Ge cage. The photoelectron spectra of LaGe*_n_*^−^ (*n* = 10–20) were simulated, and their electron affinities are reported. The average binding energies, relative stabilities, and HOMO–LUMO energy gaps of the clusters were evaluated. The UV–vis absorption spectrum of the LaGe_20_^−^ cluster was also simulated. The results demonstrate that the LaGe_20_^−^ superatom cluster with high Ih symmetry exhibits favorable optical properties, along with excellent chemical and thermodynamic stability, suggesting its potential as a promising building block for further exploration in optoelectronic-related applications.

## Figures and Tables

**Table 1:** Theoretical and experimental VDEs for Ge*_n_*^−^ (*n* = 10–20) clusters.

Isomer (*n*)	Exp. *^a^*	mPW2PLYP	Isomer (*n*)	Exp. *^a^*	mPW2PLYP
**10**	3.05	3.04	**15**	3.63	3.64
**11**	2.87	2.74	**16**	3.10	3.30
**12-1**	3.05	2.88	**17**	3.30	3.30
**12-2 *^b^***	3.05	2.50	**18**	3.13	3.10
**13**	3.40	3.23	**19**	3.38	3.31
**14**	3.25	3.13	**20**	3.45	3.37

*^a.^* The experimental values of VDE were taken from Ref. [6]. *^b^* The isomer **12-2** corresponds to the *C*_2_*ᵥ*-symmetric structure reported in ref. [1].

**Table 2:** Electronic state, average bonding energy *ABE* (eV), the charge on the La atom *Q*(La) (a.u.), and HOMO-LUMO energy gap *E*_gap_ (eV) of the GM structure of LaGe*_n_*^0/−^ (*n* = 10–20) clusters *^a^*.

	LaGe_*n*_				LaGe_*n*_^−^			
*n*	State	*ABE*	*E*_gap_	Q(La)	State	*ABE*	*E*_gap_	Q(La)
**10**	^2^A′′	3.22	3.03	0.94	^1^A	3.38	4.25	0.87
**11**	^2^A	3.23	3.55	0.76	^1^A′	3.40	4.14	0.70
**12**	^2^A	3.25	3.30	0.79	^1^A_1_	3.41	4.17	0.51
**13**	^2^A	3.28	3.15	0.88	^1^A	3.42	3.30	0.64
**14**	^2^A′	3.29	3.66	0.87	^1^A′	3.44	3.55	0.65
**15**	^2^A′	3.30	3.54	0.90	^1^A′	3.43	3.16	0.75
**16**	^2^A	3.29	3.58	0.67	^1^A′	3.44	3.41	0.56
**17**	^2^A	3.30	3.33	0.90	^1^A′	3.43	3.70	0.63
**18**	^2^A′	3.32	3.23	0.79	^1^A_1_	3.46	3.57	0.71
**19**	^2^A′	3.33	3.94	0.94	^1^A′	3.46	3.57	0.74
**20**	^2^A_1u_	3.39	2.91	−1.70	^1^A_g_	3.55	3.05	−1.62

*^a^ ABE* and *Q*(La) were obtained at the mPW2PLYP level, while *E*_gap_ was obtained at the PBE level.

**Table 3:** The theoretical VDE and adiabatic electron affinity (AEA) for LaGe*_n_*^−^ (*n* = 10–20) clusters.

Isomer (*n*)	VDE (eV)	AEA (eV)	Isomer (*n*)	VDE (eV)	AEA (eV)
**10**	3.82	2.61	**16**	3.54	3.39
**11**	3.65	2.84	**17**	3.60	3.03
**12**	3.52	2.94	**18**	3.92	3.41
**13**	2.90	2.78	**19**	3.64	3.45
**14**	3.41	2.98	**20**	4.30	4.15
**15**	3.00	2.88			

## Data Availability

Data will be made available on request.

## Supplementary Materials

The following supporting information can be downloaded at: https://www.mdpi.com/article/10.3390/molecules31152679/s1, Figure S1: The lowest energy structures and other isomers of LaGe*_n_*^0^ (*n* = 10–20) clusters after mPW2PLYP method optimization. The red and purple sphere represent La and Ge atom, respectively; Figure S2: The lowest energy structures and other isomers of anion LaGe*_n_*^−^ (*n* = 10–20) clusters after mPW2PLYP method optimization. The red and purple sphere represent La and Ge atom, respectively; Table S1: Theoretical and experimental vertical detachment energy (VDE) for Ge*_n_*^−^ (*n* = 10–20); Table S2: The NPA valence electron configuration, the charge of the La atom in LaGe*_n_*^0/−^ (*n* = 10–20).
