# Supplementary material for: Theoretical Insights into the Structures and Electronic Properties of Pure Germanium Anionic Gen− Clusters and Lanthanum-Doped Neutral and Anionic Germanium LaGen0/− Clusters (n = 10–20)
Source: Molecules. 2026 Jul 31;31(15):2679. doi: 10.3390/molecules31152679 (PMC13468060; doi:10.3390/molecules31152679)
Supplement: Supplementary file 1 [file molecules-31-02679-s001.zip › molecules-4450726-supplementary.pdf]

Supplementary Materials

Theoretical Insights into the Structures and Electronic Properties of Pure Germanium Anionic Ge_n^- Clusters and Lanthanum-Doped Neutral and Anionic Germanium $LaGe_n^{0/-}$ Clusters (n = 10–20)

Xueyan Dong [1], Zhefeng Zhang [1], Chenliang Hao [1] and Jucai Yang [1,2,*]

[1] School of Chemical Engineering, Inner Mongolia University of Technology, Hohhot 010051, China
[2] School of Resources and Environmental Engineering, Inner Mongolia University of Technology, Hohhot 010051, China
* Correspondence: yangjc@imut.edu.cn

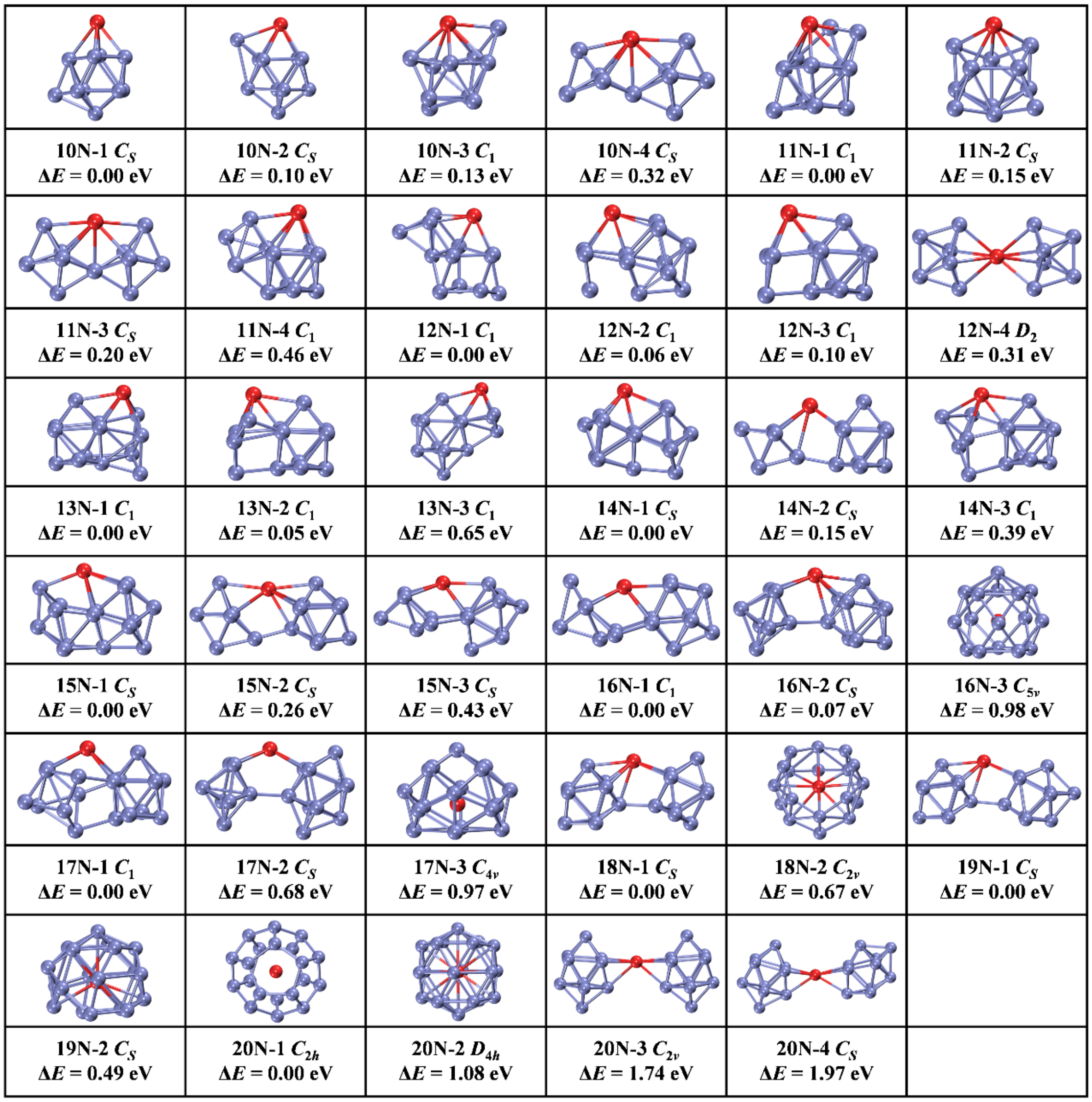

Figure S1. The lowest energy structures and other isomers of $LaGe_n^0$ (n = 10-20) clusters after mPW2PLYP method optimization. The red and purple sphere represent La and Ge atom, respectively.

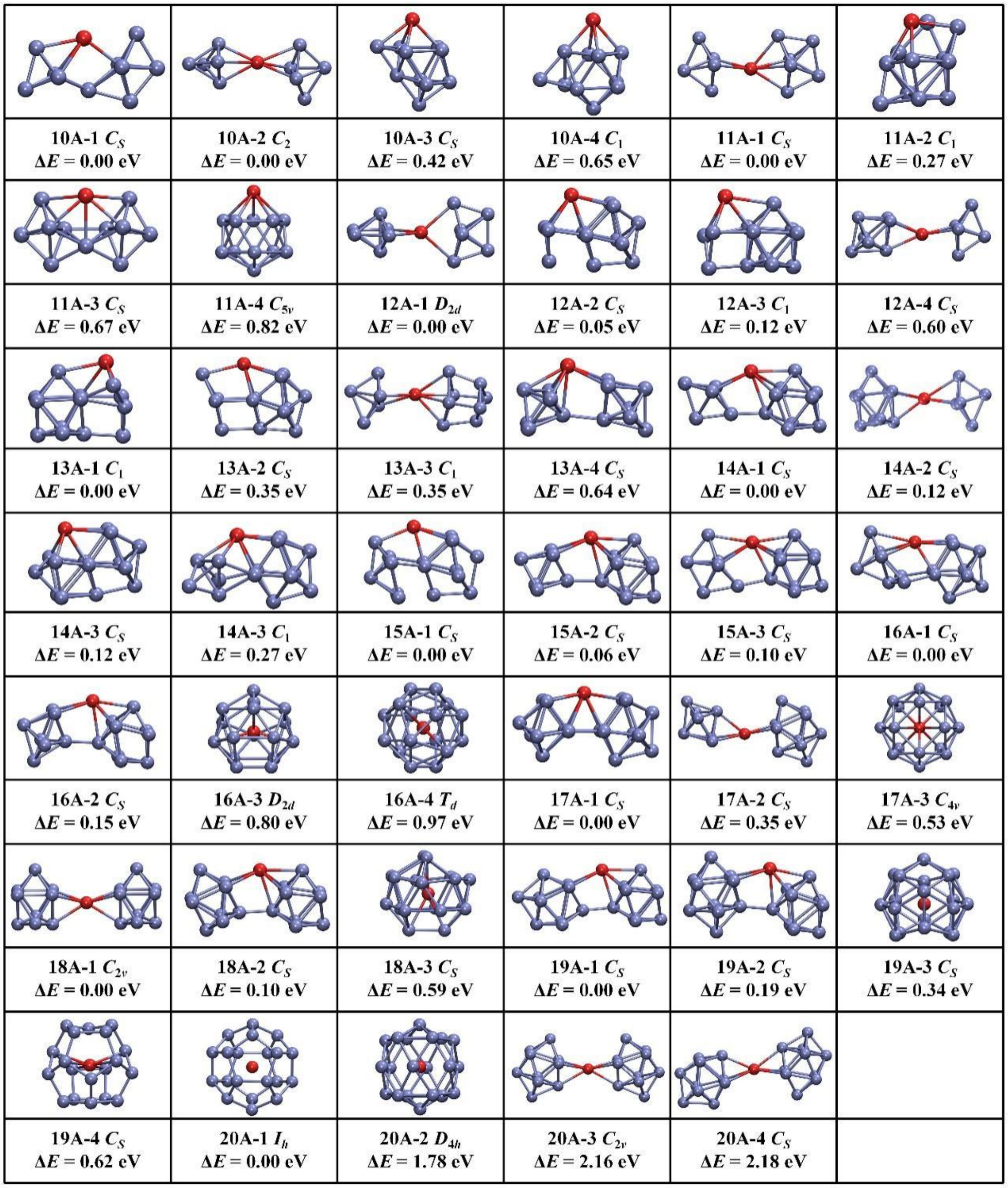

Figure S2. The lowest energy structures and other isomers of anion $LaGe_n^-$ (n = 10-20) clusters after mPW2PLYP method optimization. The red and purple sphere represent La and Ge atom, respectively.

Table S1. Theoretical and experimental vertical detachment energy (VDE) for Ge_n^- (n = 10-20)

Isomer (n)	Exp.[a]	mPW2PLYP	Isomer (n)	Exp.[a]	mPW2PLYP
**10**	3.05	3.04	**15**	3.63	3.64
**11**	2.87	2.74	**16**	3.10	3.30
**12-1**	3.05	2.50	**17**	3.30	3.30
**12-1**	3.05	2.88	**18**	3.13	3.10
**13**	3.40	3.23	**19**	3.38	3.31
**14**	3.25	3.13	**20**	3.45	3.37

[a] The experimental values of VDE were taken from Ref. [6].

Table S2. The NPA valence electron configuration, the charge of the La atom in $LaGe_n^{0/-}$ (n = 10-20)

**Clusters**	**Charge (a.u.)**	**Electron Configuration of La Atom**	**Clusters**	**Charge (a.u.)**	**Electron Configuration of La Atom**
**10N-1**	0.94	[core]$6s^{0.28}4f^{0.05}5d^{1.68}6p^{0.13}$	**10A-1**	0.87	[core]$6s^{0.30}4f^{0.03}5d^{1.42}6p^{0.38}$
**11N-1**	0.76	[core]$6s^{0.27}4f^{0.04}5d^{1.71}6p^{0.24}$	**11A-1**	0.70	[core]$6s^{0.29}4f^{0.03}5d^{1.54}6p^{0.43}$
**12N-1**	0.79	[core]$6s^{0.24}4f^{0.04}5d^{1.71}6p^{0.25}$	**12A-1**	0.51	[core]$6s^{0.28}4f^{0.03}5d^{1.65}6p^{0.49}$
**13N-1**	0.88	[core]$6s^{0.22}4f^{0.05}5d^{1.74}6p^{0.18}$	**13A-1**	0.64	[core]$6s^{0.33}4f^{0.04}5d^{1.77}6p^{0.26}$
**14N-1**	0.87	[core]$6s^{0.26}4f^{0.03}5d^{1.57}6p^{0.26}$	**14A-1**	0.65	[core]$6s^{0.30}4f^{0.04}5d^{1.67}6p^{0.36}$
**15N-1**	0.90	[core]$6s^{0.23}4f^{0.04}5d^{1.60}6p^{0.26}$	**15A-1**	0.75	[core]$6s^{0.23}4f^{0.04}5d^{1.73}6p^{0.28}$
**16N-1**	0.67	[core]$6s^{0.29}4f^{0.03}5d^{1.54}6p^{0.42}$	**16A-1**	0.56	[core]$6s^{0.30}4f^{0.04}5d^{1.60}6p^{0.45}$
**17N-1**	0.90	[core]$6s^{0.21}4f^{0.03}5d^{1.58}6p^{0.30}$	**17A-1**	0.63	[core]$6s^{0.26}4f^{0.04}5d^{1.68}6p^{0.39}$
**18N-1**	0.79	[core]$6s^{0.26}4f^{0.03}5d^{1.58}6p^{0.35}$	**18A-1**	0.71	[core]$6s^{0.22}4f^{0.04}5d^{1.70}6p^{0.37}$
**19N-1**	0.94	[core]$6s^{0.26}4f^{0.03}5d^{1.45}6p^{0.32}$	**19A-1**	0.74	[core]$6s^{0.30}4f^{0.03}5d^{1.61}6p^{0.34}$
**20N-1**	-1.70	[core]$6s^{0.31}4f^{0.03}5d^{2.96}6p^{1.07}$	**20A-1**	-1.62	[core]$6s^{0.30}4f^{0.03}5d^{2.91}6p^{1.06}$
